# Nanometer-size hard magnetic ferrite exhibiting high optical-transparency and nonlinear optical-magnetoelectric effect

**DOI:** 10.1038/srep14414

**Published:** 2015-10-06

**Authors:** Shin-ichi Ohkoshi, Asuka Namai, Kenta Imoto, Marie Yoshikiyo, Waka Tarora, Kosuke Nakagawa, Masaya Komine, Yasuto Miyamoto, Tomomichi Nasu, Syunsuke Oka, Hiroko Tokoro

**Affiliations:** 1 Department of Chemistry, School of Science, The University of Tokyo, 7-3-1 Hongo, Bunkyo-ku, Tokyo 113-0033, Japan; 2 CREST, JST, K’s Gobancho, 7 Gobancho, Chiyoda-ku, Tokyo 102-0076, Japan; 3 Division of Materials Science, Faculty of Pure and Applied Sciences, University of Tsukuba, 1-1-1 Tennodai, Tsukuba, Ibaraki 305-8577, Japan

## Abstract

Development of nanometer-sized magnetic particles exhibiting a large coercive field (*H*_c_) is in high demand for densification of magnetic recording. Herein, we report a *single-nanosize* (i.e., less than ten nanometers across) hard magnetic ferrite. This magnetic ferrite is composed of ε-Fe_2_O_3_, with a sufficiently high *H*_c_ value for magnetic recording systems and a remarkably high magnetic anisotropy constant of 7.7 × 10^6^ erg cm^−3^. For example, 8.2-nm nanoparticles have an *H*_c_ value of 5.2 kOe at room temperature. A colloidal solution of these nanoparticles possesses a light orange color due to a wide band gap of 2.9 eV (430 nm), indicating a possibility of transparent magnetic pigments. Additionally, we have observed magnetization-induced second harmonic generation (MSHG). The nonlinear optical-magnetoelectric effect of the present polar magnetic nanocrystal was quite strong. These findings have been demonstrated in a simple iron oxide, which is highly significant from the viewpoints of economic cost and mass production.

Magnetic materials have been used for a whole host of applications: magnetic recording media, permanent magnets, electromagnetic wave absorbers, magnetic fluid, drug delivery, to name a few^1^,^2^,^3^,^4^,^5^,^6^,^7^,^8^. From the viewpoint of densification of magnetic recording media, development of a nanometer-sized magnetic particle (less than 10 nm in diameter) with a large coercive field (*H*_c_) is the logical next step. In magnetic recording systems^9^,^10^,^11^, such as hard drives or magnetic recording tapes, the required *H*_c_ value for writing and reading is *ca*. 3 kOe. A larger *H*_c_ is necessary for future magnetic recording systems, such as bit-patterned media or heat-assisted magnetic recording. Materials exhibiting multiferroic properties are drawing increasing attention^12^,^13^,^14^,^15^,^16^,^17^ as electrically assisted magnetic recording media^13^. In addition, development of an optically transparent magnet is highly desirable for new applications, such as transparent electromagnetic wave-absorbing windows or magnetic color pigments for printers. In light of the above requirements, epsilon iron oxide ε-Fe_2_O_3_ is an attractive material because it exhibits a large *H*_c_ value at room temperature^7^,^18^,^19^,^20^,^21^,^22^,^23^,^24^,^25^. In the present work, we develop a synthetic method for the preparation of *single-nanosize* ε-Fe_2_O_3_ spherical particles. The resulting nanomagnets satisfy the required *H*_c_ value for magnetic recording applications mentioned above. In addition, they exhibit magnetization-induced second harmonic generation (MSHG), with a strong magnetoelectric (ME) effect. The color of this series is very light, and the absorption coefficient is small in the visible region. In this work, we report the synthesis procedure, the crystal structure, the particle sizes, and magnetic properties of nanometer-size ε-Fe_2_O_3_ nanoparticles. Furthermore, we present first-principles calculations for the optical band gap, the spontaneous electric polarization of the polar crystal, and the nonlinear optical-ME effect.

## Results and Discussion

Nanometer-size ε-Fe_2_O_3_ was prepared from a precursor, in which ferrihydrite Fe_10_O_14_(OH)_2_ nanoparticles were embedded in SiO_2_ matrix. The details of the synthetic procedure are described in the Methods section. In this report, we describe 18 samples sintered at a large range of temperatures: 250 °C (**S-250**), 500 °C (**S-500**), 731 °C (**S-731**), 902 °C (**S-902**), 924 °C (**S-924**), 951 °C (**S-951**), 979 °C (**S-979**), 1002 °C (**S-1002**), 1020 °C (**S-1020**), 1032 °C (**S-1032**), 1044 °C (**S-1044**), 1061 °C (**S-1061**), 1063 °C (**S-1063**), 1104 °C (**S-1104**), 1142 °C (**S-1142**), 1198 °C (**S-1198**), 1213 °C (**S-1213**), and 1295 °C (**S-1295**).

**Figure 1:**
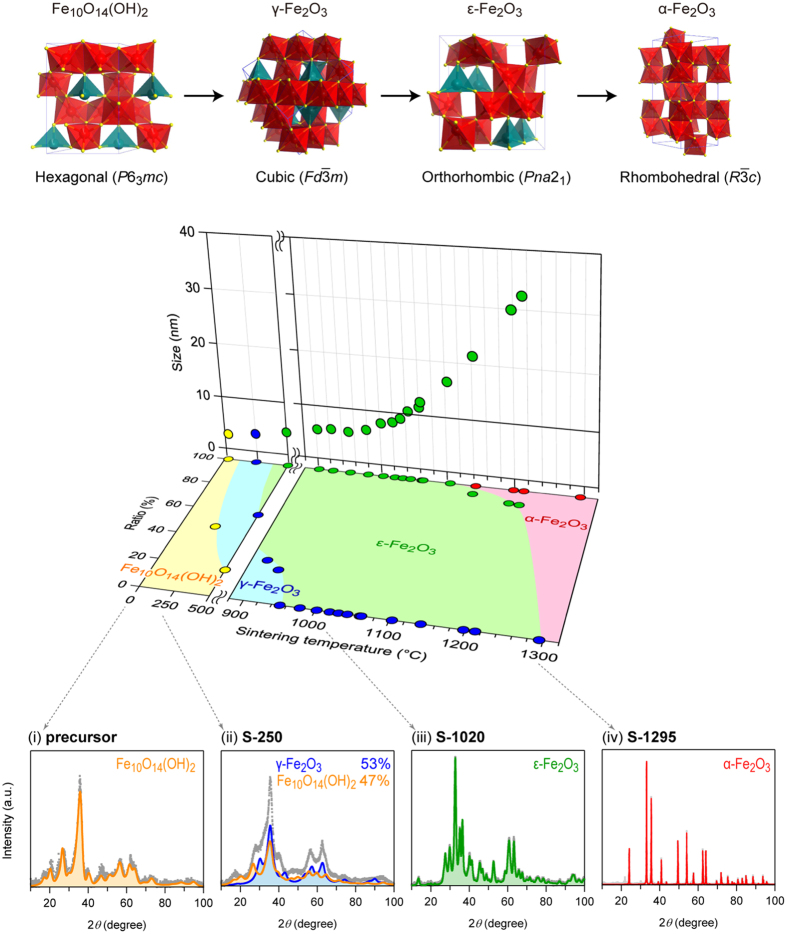
Phase transformation of the crystal structure in the sintering process. (Top) Crystal structures of Fe_10_O_14_(OH)_2_, γ-Fe_2_O_3_, ε-Fe_2_O_3_, and α-Fe_2_O_3_. Red and blue polyhedra in the crystal structure indicate the octahedral and tetrahedral Fe sites, respectively. Yellow balls represent oxygen atoms. (Middle) Sintering temperature dependence of the particle size (vertical panel) and phase diagram showing the phase ratio versus sintering temperature (horizontal panel). (Bottom) XRPD patterns of (i) the precursor Fe_10_O_14_(OH)_2_, (ii) **S-250**, (iii) **S-1020**, and (iv) **S-1295**. The silica matrix in the precursor and the remaining silica in **S-1295** are extracted. Grey dots, and the orange, blue, green, and red areas indicate the observed pattern and the calculated patterns of Fe_10_O_14_(OH)_2_, γ-Fe_2_O_3_, ε-Fe_2_O_3_, and α-Fe_2_O_3_, respectively.

X-ray powder diffraction (XRPD) patterns of the sintered samples and the precursor are shown in Fig. 1 and Supplementary Fig. S1. The XRPD pattern of the precursor shows Fe_10_O_14_(OH)_2_ having hexagonal crystal structure (space group *P*6_3_*mc*, *a* = 6.04 Å, *c* = 8.75 Å). With increasing sintering temperature, Fe_10_O_14_(OH)_2_ begins to transform into γ-Fe_2_O_3_ (cubic, *Fd3–m*, *a* = 8.37 Å) around 250 °C. As the sintering temperature is raised, γ-Fe_2_O_3_ transforms into ε-Fe_2_O_3_ around 500 °C. Above 951 °C, the XRPD patterns for **S-951**–**S-1104** show a pure ε-Fe_2_O_3_ phase (orthorhombic, *Pna*2_1_, *a* = 5.09 Å, *b* = 8.80 Å, and *c* = 9.48 Å for **S-1020**) (Supplementary Table S1). Above 1142 °C, a small amount of α-Fe_2_O_3_ is detected (rhombohedral, *R*3–*c*, *a* = 5.04 Å, and *c* = 13.75 Å for **S-1295**). In this synthetic method, pure ε-Fe_2_O_3_ is obtained in a surprisingly wide sintering temperature range. The range is wider than those of the previously reported methods, including a combination of reverse-micelle and sol–gel methods^20^ and impregnation method using mesoporous silica^7^.

The transmission electron microscopy (TEM) image of the precursor shows that the particle size (*d*) of Fe_10_O_14_(OH)_2_ nanoparticles is 2.8 ± 0.5 nm. In the sintering temperature range up to 700 °C, which is the region of the Fe_10_O_14_(OH)_2_ → γ-Fe_2_O_3_ transition, the *d* value is almost constant around 3–4 nm (Fig. 1, middle). As the sintering temperature increases further, from 750 °C to 924 °C, which is the region of the γ-Fe_2_O_3_ → ε-Fe_2_O_3_ transition, the *d* value gradually increases, and pure ε-Fe_2_O_3_ is formed. The *d* values of the ε-Fe_2_O_3_ samples are as follows: 5.6 ± 1.6 nm (**S-951**), 6.3 ± 1.7 nm (**S-979**), 7.8 ± 2.7 nm (**S-1002**), 8.2 ± 2.7 nm (**S-1020**), 9.0 ± 2.4 nm (**S-1032**), 10.5 ± 3.3 nm (**S-1044**), 11.4 ± 3.8 nm (**S-1061**), 12.4 ± 3.7 nm (**S-1063**), and 16.5 ± 4.7 nm (**S-1104**). The TEM images of all of the samples are shown in Supplementary Fig. S2.

**Figure 2:**
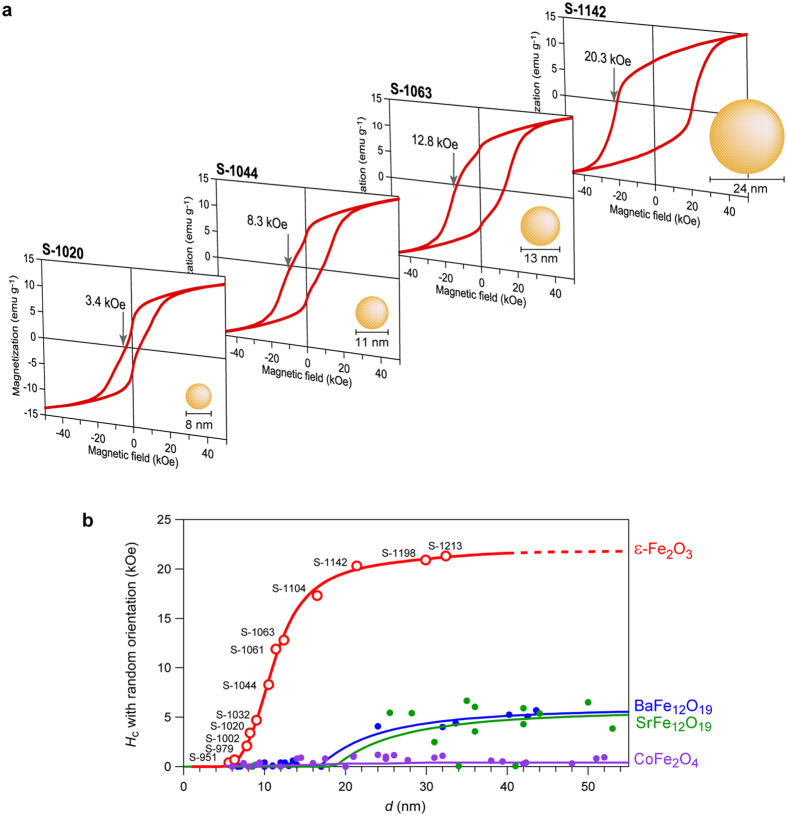
Particle size dependence of the magnetic properties. (**a**) Magnetic hysteresis loops of **S-1020**, **S-1044**, **S-1063**, and **S-1142** measured at 300 K with an illustration of the average particle size. (**b**) *H*_c_ value at 300 K with random orientation versus *d* plot. The red line is a guide for the eye, which was drawn based on the *d* dependence equation of *H*_c_, taking into account the random orientation and particle size distribution. The *d*_p_ (superparamagnetic limit) value was calculated to be 7.5 nm. The *H*_c_ versus *d* plots at room temperature of BaFe_12_O_19_ (blue), SrFe_12_O_19_ (green), and CoFe_2_O_4_ (purple) are also shown.

The magnetic hysteresis loops of ε-Fe_2_O_3_ for **S-951**–**S-1198** with random orientation at 300 K show that the *H*_c_ values are 0.4 kOe (**S-951**), 0.7 kOe (**S-979**), 2.1 kOe (**S-1002**), 3.4 kOe (**S-1020**), 4.7 kOe (**S-1032**), 8.3 kOe (**S-1044**), 11.9 kOe (**S-1061**), 12.8 kOe (**S-1063**), 17.3 kOe (**S-1104**), 20.3 kOe (**S-1142**), and 20.9 kOe (**S-1198**) (Fig. 2a, Supplementary Fig. S3). The magnetization versus temperature plots for **S-951**–**S-1104** are shown in Supplementary Fig. S4. As shown in the *H*_c_ versus *d* plot of Fig. 2b, the *H*_c_ value decreases towards zero with decreasing *d*. In Fig. 2b, the particle size dependences of the *H*_c_ values of BaFe_12_O_19_, SrFe_12_O_19_, and CoFe_2_O_4_ reported so far are also plotted for reference (Supplementary Fig. S5).

Let us consider the *d* dependence of the *H*_c_ value. The synthesized ε-Fe_2_O_3_ nanoparticles have a size distribution. A theoretical equation for superparamagnetic limit (*d*_p_) of nanoparticles with random orientation considering a size distribution was derived by H. Pfeiffer^26^ (see Methods section). Based on this equation, the *H*_c_ versus *d* plot was fitted, and the *d*_p_ value was estimated to be 7.5 nm (Fig. 2b).

**Figure 3:**
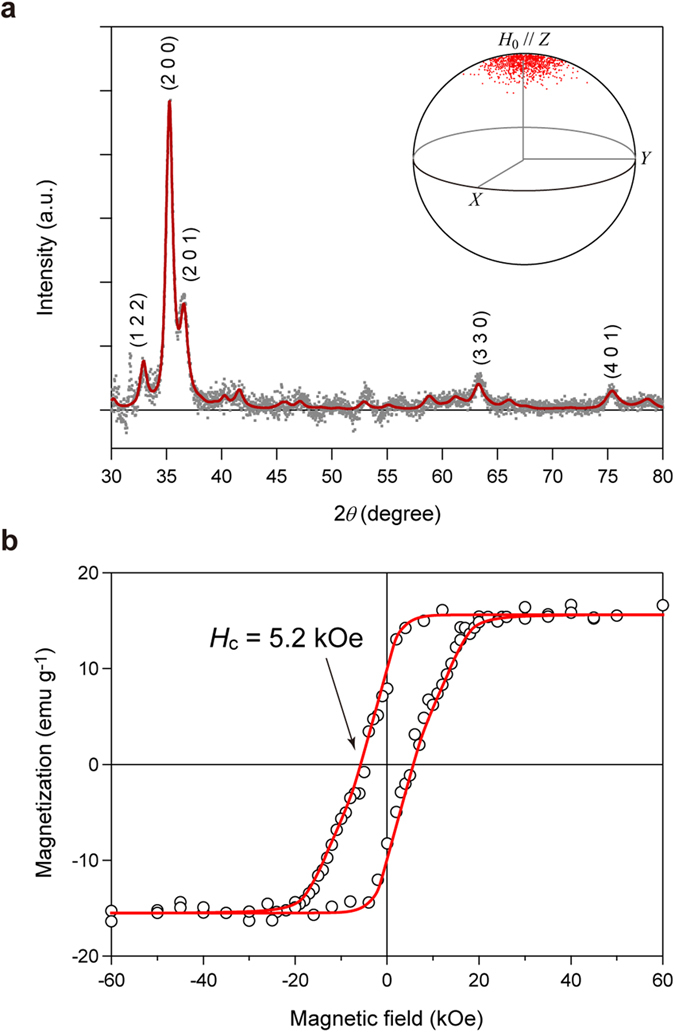
Crystallographically oriented nanometer-sized ε-Fe_2_O_3_. (**a**) XRPD pattern of the crystallographically oriented **S-1020** nanocrystal (average particle size = 8.2 nm) film. Grey dots and red line represent the observed and calculated patterns, respectively. The inset is the unit sphere illustration of the 3D distribution of the direction of the crystallographic *a*-axis of ε-Fe_2_O_3_ nanoparticles shown by red dots. The magnetic field was applied along the *Z* axis of the unit sphere. (**b**) The magnetic hysteresis loop of the crystallographically oriented **S-1020** nanocrystal film measured in the applied magnetic field (*H*_0_) parallel to the easy-axis at 300 K. The red line is a guide to the eye.

In addition, we prepared oriented nanocrystal thin film, which was obtained by dispersing the nanocrystals in a vehicle matrix under external magnetic field. The XRPD pattern of the **S-1020** shows that the nanocrystals oriented along the crystallographic *a*-axis, perpendicular to the film (Fig. 3a). The magnetic hysteresis loop at 300 K shows that the ε-Fe_2_O_3_ nanocrystal with the size of 8.2 nm exhibits a large coercive field of 5.2 kOe (Fig. 3b, Supplementary Fig. S6), which meets the *H*_c_ value criterion for magnetic memory media. The magnetic measurement of the oriented **S-1142** film was also conducted. The *H*_c_ value of the magnetic hysteresis loop and the natural resonance frequency of 182 GHz, reported in our previous works^7^,^23^, indicate that the magnetic anisotropy constants of *K*_a_ and *K*_b_ in orthorhombic symmetry are 7.7 × 10^6^ erg cm^−3^ and 1.2 × 10^6^ erg cm^−3^, respectively. Therefore, it is clarified that ε-Fe_2_O_3_ has remarkably high magnetic anisotropy compared with other ferrites such as BaFe_12_O_19_. The origin of such a small *d*_p_ value and large *K* values can be explained by the following factors: (i) a strong magnetic anisotropy due to non-zero orbital angular momentum, *L* ≠ 0, through a strong hybridization between Fe and O, and (ii) remnants of the magnetic anisotropy due to the *polar crystal* structure.

**Figure 4:**
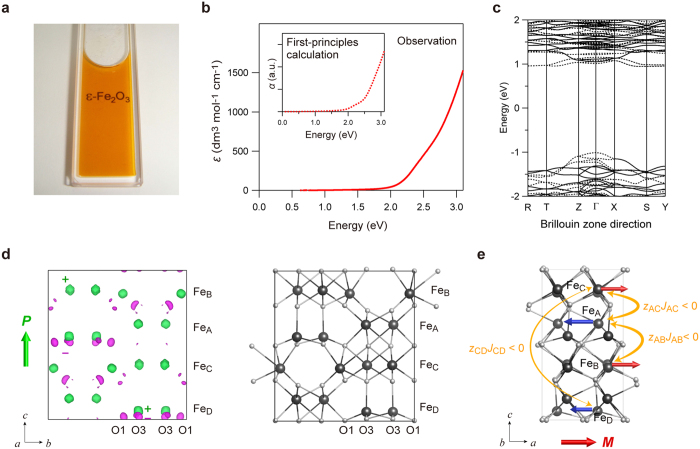
Transparency, optical band gap, and spontaneous electric polarization of ε-Fe_2_O_3_ by UV-vis absorption spectra measurement and first-principles calculation. (**a**) Photograph of the colloidal solution of **S-1020** nanocrystal with an average particle size of 8.2 nm, whose concentration is 1 × 10^−2^ mol dm^−3^. The solution has a light orange color. (**b**) Observed UV-vis absorption spectrum of **S-1020** colloidal solution shown by the molar absorption coefficient (*ε*). The inset shows the calculated optical spectrum obtained from the first-principles calculation. The left axis is the absorption coefficient (*α*). (**c**) The band structure of ε-Fe_2_O_3_ near the Fermi energy. Solid and dotted black lines indicate α and β spins, respectively. (**d**) The difference charge density map, which shows the difference between the charge density of ε-Fe_2_O_3_ and those of the neutral Fe and O atoms, obtained by the first-principles calculation (left). Green and magenta surfaces in the difference charge density map show the isosurface levels of +0.385*e* Å^−3^ and –0.252*e* Å^−3^, respectively. The green arrow shows that the spontaneous electric polarization (*P*) of ε-Fe_2_O_3_ is along the crystallographic *c*-axis. The crystal structure of ε-Fe_2_O_3_ (right). The grey and white balls in the crystal structure show the Fe and O atoms, respectively. (**e**) Sublattice magnetizations of Fe_A_–Fe_D_ shown by the arrow on the crystal structure of ε-Fe_2_O_3_. Red and blue arrows on the Fe sites indicate the sublattice magnetizations. The yellow curved arrows express the *zJ* values, where *z* is the number of exchange pathways and *J* is the superexchange interaction constant, with antiferromagnetic superexchange interaction between the Fe sites (*z*_AB_ *J*_AB_ < 0, *z*_AC_ *J*_AC_ < 0, *z*_CD_ *J*_CD_ < 0), and the thickness of the arrows indicate that the magnitude of *zJ* is 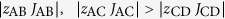. The red arrow below the crystal structure shows that the magnetic polarization (*M*) of ε-Fe_2_O_3_ is along the crystallographic *a*-axis.

The colloidal solution of the **S-1020** nanocrystal, which was used for the oriented nanocrystal film mentioned above, is highly transparent, with a light orange color (Fig. 4a). From the ultraviolet-visible (UV-vis) absorption spectrum, the molar absorption coefficient (*ε*) was only 400 dm^3^ mol^−1^ cm^−1^ at 500 nm (Fig. 4b). (Photon energy × absorption)^2^ versus photon energy was well fitted by a wide band gap of 2.9 eV (430 nm) accompanied by a weak optical transition at 2.4 eV (520 nm), indicating that this material has high transparency due to a wide band gap of 2.9 eV. Compared to γ-Fe_2_O_3_ nanoparticles, which have been reported to possess light color^27^, the present material exhibits a larger band gap and higher transparency.

To understand such a high transparency, the optical band gap of ε-Fe_2_O_3_ was evaluated by first-principles calculation using the Vienna *ab initio* simulation package (VASP) program (see Methods). The band structure near the Fermi energy is shown in Fig. 4c, which shows an optical transition with a band gap of 2.36 eV, through a direct transition, and a weak transition at 2.02 eV. The calculated optical absorption spectrum reproduces well the experimental spectrum (Fig. 4b, inset).

The crystal structure of ε-Fe_2_O_3_ with *Pna*2_1_ space group is that of a polar crystal, and therefore, spontaneous electric polarization should be generated. Here, the magnitude of spontaneous electric polarization of ε-Fe_2_O_3_ and its origin are investigated by a first-principles calculation. The results show that electric polarization exists along the crystallographic *c*-axis with a value of 1.0 × 10^−1^ C m^−2^, which is large compared with other polar materials^28^. The difference charge density map, which is the difference between the charge density of ε-Fe_2_O_3_ and those of the neutral Fe and O atoms, indicates that the positive charge is distributed on the Fe atoms, and negative charge is distributed on the O atoms, as shown in Fig. 4d. The negative charge, especially, is concentrated on the O1 and O3 atoms around the tetrahedral Fe_D_ site, indicating that the electric polarization along the *c*-axis at Fe_D_O_4_ is the main contribution to the pyroelectric property of ε-Fe_2_O_3_.

The magnetism of ε-Fe_2_O_3_ is considered to be collinear ferrimagnetism^18^,^19^, in which the sublattice magnetizations of Fe_B_ and Fe_C_ are antiparallel to those of Fe_A_ and Fe_D_. Based on the molecular-field (MF) theory^29^, the magnetic structure of ε-Fe_2_O_3_ can be understood from the product value between the superexchange interaction (*J*) and the number of exchange pathways (*z*). The *zJ* value of the tetrahedral Fe_D_ site is smaller than those of octahedral Fe_A_–Fe_C_ sites, and hence, thermal fluctuation on Fe_D_ sublattice magnetization is larger than Fe_A_–Fe_C_ sublattice magnetizations, inducing ferrimagnetism along the crystallographic *a*-axis at room temperature (Fig. 4e).

**Figure 5:**
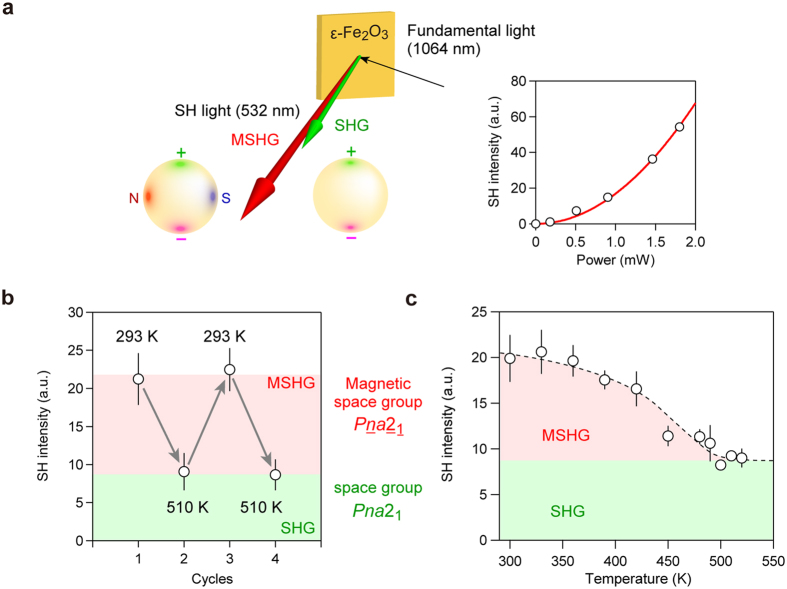
SHG and MSHG effects on ε-Fe_2_O_3_. (**a**) The optical configuration of SHG and MSHG measurements. The orange square shows the position of the ε-Fe_2_O_3_ sample. A 1064-nm laser light (black arrow) irradiates the sample, and 532-nm SH output light is observed. The green arrow shows the SHG when the sample is in the magnetic-disordering state, while the red arrow shows the MSHG when the sample is in the magnetic-ordering state. The yellow spheres show the schematic illustrations of ε-Fe_2_O_3_ nanoparticle in the magnetic-ordering state with *Pna*2_1_ magnetic space group (left) and the magnetic-disordering state with *Pna*2_1_ space group (right). Only the electric polarization exists in the magnetic-disordering state, where the positive and negative charge are shown by green and magenta. In the magnetic-ordering state, the magnetic polarization appears perpendicular to the electric polarization, which is shown by the N-pole in red and S-pole in blue. The bottom right figure shows the SH intensity of **S-1061** versus fundamental light intensity at room temperature. The red line represents the fitted curve based on SH intensity ∝ (fundamental light power)^2^. (**b**) Repetitive switching of SH intensity between SHG and MSHG by changing the temperature between above and below *T*_C_. (**c**) SH intensity versus temperature plot of **S-1061**.

From the aforementioned electronic and magnetic calculations, ε-Fe_2_O_3_ has both electric polarization (//*c*-axis) and magnetic polarization (//*a*-axis). To investigate the magnetoelectric coupling effect between these two polarizations^30^,^31^,^32^, we measured the MSHG effect using the powder-form sample of **S-1061**. When a fundamental femtosecond laser (1064 nm) was put into the sample at 300 K, 532-nm output light was observed (Fig. 5a, Supplementary Fig. S7). Since the intensity of the 532-nm output light increased with the square of the input fundamental light intensity, the observed 532-nm light was due to second harmonic generation (SHG). By switching of the magnetic state between order and disorder, we were able to change the second harmonic (SH) intensity (*I*_SH_) repeatedly (Fig. 5b). We have also investigated the temperature dependence of *I*_SH_, which turned out to be nearly constant between 520 and 490 K, while gradually increasing below 490 K (Fig. 5c). The *I*_SH_ value at 300 K was 2.2 times larger than the average *I*_SH_ at 520 K. Such a temperature dependence of *I*_SH_ corresponds to the magnetization versus temperature plot with a magnetic phase transition temperature (*T*_C_) of 490 K (Supplementary Fig. S4), indicating that the enhancement on *I*_SH_ is caused by magnetic ordering, that is, MSHG effect is observed.

SH polarization is described by *P*_SH_ = *χ*^(2)^*E*(*ω*)*E*(*ω*), where *P*_SH_ and *χ*^(2)^ are the SH polarization and SH susceptibility tensor, and *E*(*ω*) and *ω* are the electric field and angular frequency of the input fundamental light, respectively (see Methods). The *I*_SH_ value is related to the crystallographic term 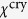 and the magnetic term 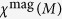 by the equation of 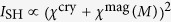. Below *T*_C_, 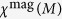 operates, and then, *I*_SH_ is enhanced depending on the magnetization value. From the SHG and MSHG results, the nonlinear optical-ME effect of these nanocrystals is quite strong. That is, the magnetic polarization and electric polarization are strongly correlated to each other in ε-Fe_2_O_3_.

In summary, we have developed a synthetic method for a single-nanosize ε-Fe_2_O_3_ magnet. In particular, the *H*_c_ value of ε-Fe_2_O_3_ with a diameter of 8 nm is 5 kOe, sufficient for magnetic recording systems, where the necessary spec is 3 kOe. Magnetic anisotropy constant approaches 7.7 × 10^6^ erg cm^−3^, which is over two times as large as those of BaFe_12_O_19_ (*K* = 3.0 × 10^6^ erg cm^−3^) and SrFe_12_O_19_ (*K* = 3.5 × 10^6^ erg cm^−3^). The present single-nanosize magnetic material may contribute to the high-density magnetic recording technology, for example, LTO-8 of the next-generation magnetic recording tapes, as well as hard disc drives for computers^4^,^5^,^9^,^10^,^11^. While this magnitude of magnetization can be detected by high-sensitive reading heads, we are in the process of improving the magnetization value of this series by a double using the approach of metal-substitution procedure. The ε-Fe_2_O_3_ nanomagnet also exhibits spontaneous electric polarization, originating from its polar crystal structure, and an optical-magnetoelectric effect between electric polarization (//*c*-axis) and magnetic polarization (//*a*-axis), with an MSHG effect. Surprisingly, this nonlinear optical-ME effect is strong. Importantly, this material possesses high optical transparency with a wide band gap. A transparent magnet will open possibilities for new industrial applications, e.g. transparent electromagnetic wave absorbing windows and magnetic color pigments. Such high performance was achieved in a simple iron oxide, which is significant, since iron oxides are ecofriendly and low cost. Therefore, this material can be reasonably expected to be scalable for industrial applications.

## Methods

### Materials

Nanometer-size ε-Fe_2_O_3_ was prepared from a precursor, where Fe_10_O_14_(OH)_2_^33^ with particle size of 2.8 nm was embedded in SiO_2_. The precursor was sintered at 250 °C–1295 °C for four hours in air, to obtain iron oxide in SiO_2_ matrix. The SiO_2_ matrix was then removed by chemical etching using a NaOH aqueous solution.

### Physical property measurements

TEM images were acquired with JEM 2000EX. The 2*θ*–*θ* scan XRPD measurements were performed using Rigaku Ultima IV and Rigaku RINT2100 with Cu Kα radiation (*λ* = 1.5418 Å) at 293 K. Rietveld analyses were performed using the PDXL program of RIGAKU. The magnetic properties were measured using a superconducting quantum interference device (SQUID) magnetometer (Quantum Design, MPMS 7). UV-vis spectra were recorded on JASCO V-670 spectrometer.

### Particle size dependence of the coercive field

In the case of nanoparticles with a random orientation considering a size distribution, the *d* value dependence of the *H*_c_ value is described by


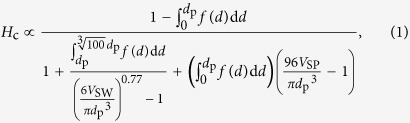


where *V*_SP_ and *V*_SW_ are the mean volumes of superparamagnetic particles and particles with sizes between *d*_p_ and 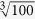
*d*_p_, respectively, and *f*(*d*) is the particle size distribution^26^,^34^. The Gaussian function is used as *f*(*d*) in the present analysis.

### First-principles calculation of electronic structure, optical spectrum, and spontaneous electric polarization

First-principles calculations of ε-Fe_2_O_3_ was carried out using the VASP program with the plane-wave projector augmented wave (PAW) method. The spin-polarized density functional theory (DFT) was used as the basis. Approximation of the exchange-correlation functional was done using the generalized gradient approximation (GGA) parameterized by Perdew-Burke-Ernzerhof (PBE).

### Experimental details for SHG measurement

For the SHG measurement, incident 1064-nm light was generated by an optical parametric amplifier (Clark-MXR, Vis-OPA; pulse width 190 fs; repetition, 1 kHz) pumped by a frequency-doubled Ti:sapphire laser (Clark-MXR, CPA-2001; wavelength, 775 nm; pulse width 150 fs; repetition, 1 kHz). The incident light was irradiated onto the sample at the angle of 20°. The detection of the reflected SH light (532 nm) was performed by a photomultiplier tube (Hamamatsu R329-02), after passing through color filters.

### SHG and MSHG tensors

SH polarization is described by *P*_SH*,i*_ = *χ*_*ijk*_^(2)^*E*_*j*_(*ω*)*E*_*k*_(*ω*), where *P*_SH*,i*_ and *χ*_*ijk*_^(2)^ are the SH polarization and SH susceptibility tensor, and the subscripts *i*, *j*, and *k*, refer to the axes of the sample. The space group *Pna*2_1_ of ε-Fe_2_O_3_ has a crystallographic term 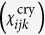 in the second-order nonlinear susceptibility^35^. When the nanoparticles are oriented by electric and magnetic fields as (electric polarization) //*z*-axis (*c*-axis) and (magnetic polarization) //*x*-axis (*a*-axis), the nonzero 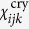 elements in *χ*_*ijk*_^(2)^ are 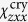, 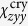, 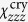, 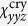 and 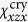. Additionally, below *T*_C_, the magnetic polarization generates a magnetic term 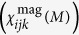 in the *Pna*2_1_ magnetic space group, that is, 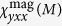, 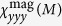, 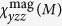, 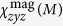 and 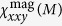. The nonzero tensor elements in *χ*_*ijk*_^(2)^ are described by the sum of 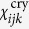 and 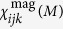 by the following tensor.


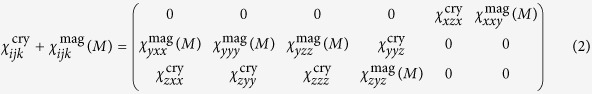


## Additional Information

**How to cite this article**: Ohkoshi, S. *et al.* Nanometer-size hard magnetic ferrite exhibiting high optical-transparency and nonlinear optical-magnetoelectric effect. *Sci. Rep.* **5**, 14414; doi: 10.1038/srep14414 (2015).

## Supplementary Material

Supplementary Information
