# Supplementary material for: Nanometer-size hard magnetic ferrite exhibiting high optical-transparency and nonlinear optical-magnetoelectric effect
Source: Sci Rep. 2015 Oct 6;5:14414. doi: 10.1038/srep14414 (PMC4594123; doi:10.1038/srep14414)
Supplement: Supplementary Information [file srep14414-s1.pdf]

Nanometer-size hard magnetic ferrite exhibiting high optical-transparency and nonlinear optical-magnetoelectric effect

Shin-ichi Ohkoshi,[1,2,]* Asuka Namai,[1] Kenta Imoto,[1] Marie Yoshikiyo,[1] Waka Tarora,[1] Kosuke Nakagawa,[1] Masaya Komine,[1] Yasuto Miyamoto,[1] Tomomichi Nasu,[1] Syunsuke Oka,[1] and Hiroko Tokoro[1,3]

[1]*Department of Chemistry, School of Science, The University of Tokyo,*
7-3-1 Hongo, Bunkyo-ku, Tokyo 113-0033, Japan
[2]*CREST, JST, K's Gobancho, 7 Gobancho, Chiyoda-ku, Tokyo 102-0076, Japan*
[3]*Division of Materials Science, Faculty of Pure and Applied Sciences, University of Tsukuba,*
1-1-1 Tennodai, Tsukuba, Ibaraki 305-8577, Japan

*To whom correspondence should be addressed
E-mail: ohkoshi@chem.s.u-tokyo.ac.jp

Contents: **Page**

1.	Crystal structure analyses	Fig. S1, Table S1	S2–S4
2.	Morphology	Fig. S2	S5
3.	Magnetic hysteresis loops	Fig. S3	S6–S7
4.	Magnetization versus temperature curves	Fig. S4	S8
5.	Particle size dependences of the H_c values	Fig. S5	S9
6.	Crystallographically oriented nanocrystal film	Fig. S6	S10
7.	SHG measurement	Fig. S7	S11

§ 1. Crystal structure analyses

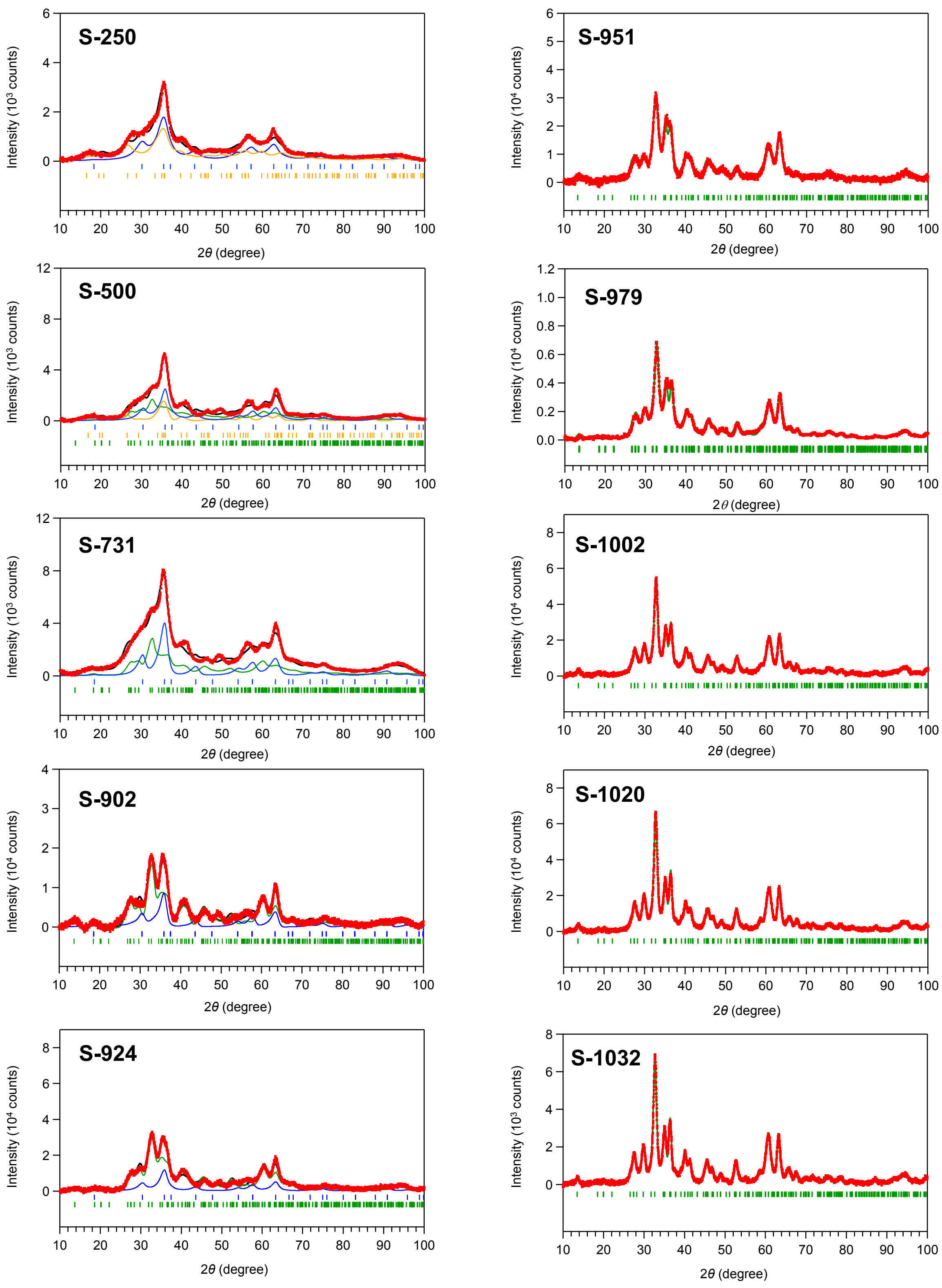

Figure S1-1. XRPD patterns and Rietveld analyses for **S-250**, **S-500**, **S-731**, **S-902**, **S-924**, **S-951**, **S-979**, **S-1002**, **S-1020**, and **S-1032**. Red dots and black lines are the observed patterns and calculated patterns, respectively. Colored lines and bars represent the calculated patterns and Bragg reflection positions for $Fe_{10}O_{14}(OH)_2$ (orange), γ-Fe_2O_3 (blue), and ε-Fe_2O_3 (green).

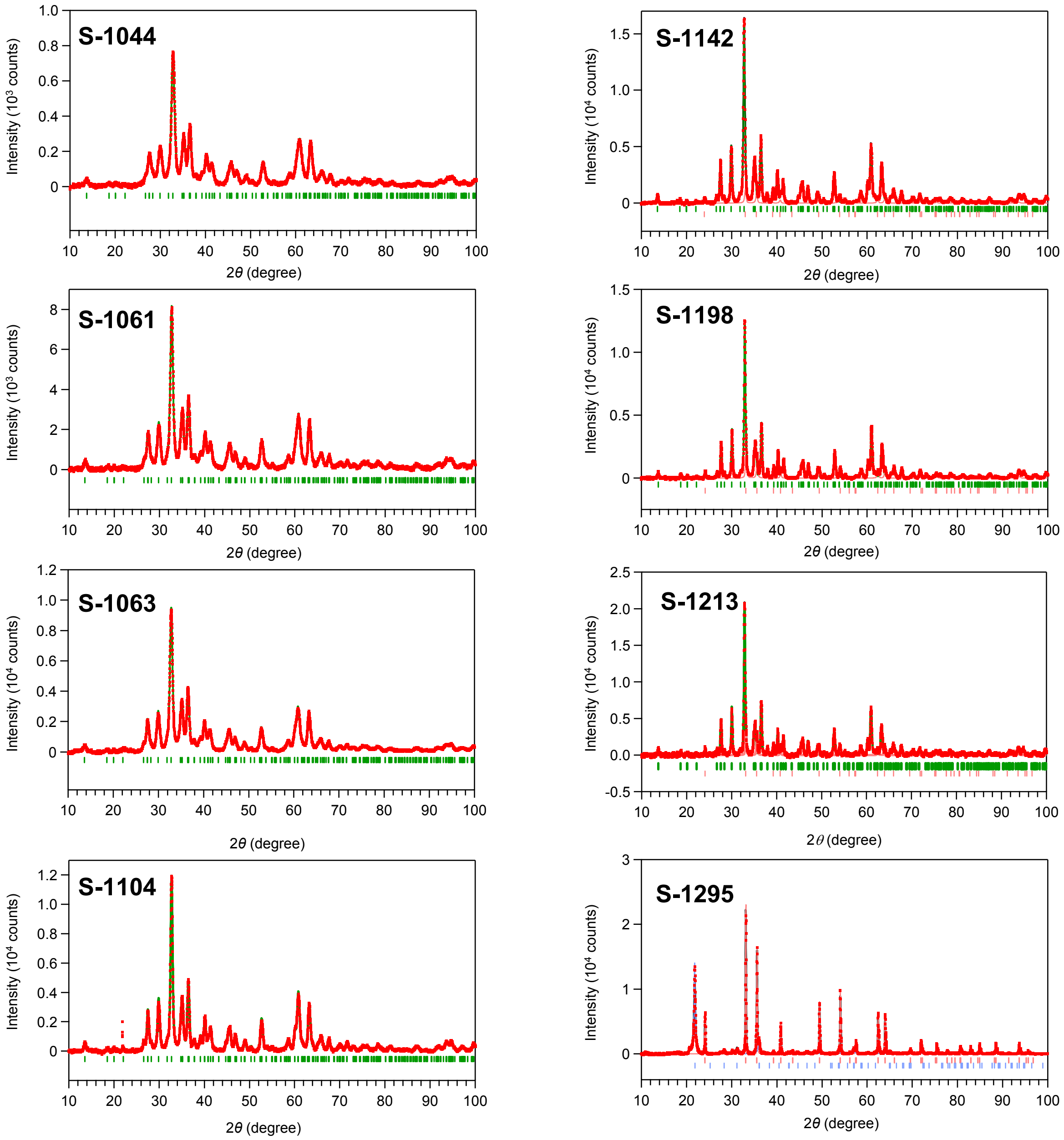

Figure S1-2. XRPD patterns and Rietveld analyses for **S-1044**, **S-1061**, **S-1063**, **S-1104**, **S-1142**, **S-1198**, **S-1213**, and **S-1295**. Red dots and black lines are the observed patterns and calculated patterns, respectively. Colored lines and bars represent the calculated patterns and Bragg reflection positions for ε-Fe_2O_3 (green), α-Fe_2O_3 (pink), and SiO_2 (light blue).

Table S1. Lattice parameters of ε-Fe_2O_3 in **S-951**, **S-979**, **S-1002**, **S-1020**, **S-1032, S-1044**, **S-1061**, **S-1063**, **S-1104**, **S-1142**, **S-1198**, and **S-1213** refined by Rietveld analyses of the XRPD patterns.

**Sample**	*a* (Å)	*b* (Å)	*c* (Å)	*V* ($Å^3$)
**S-951**	5.0800(9)	8.800(2)	9.481(3)	423.8(2)
**S-979**	5.0798(15)	8.800(6)	9.485(2)	424.0(2)
**S-1002**	5.0841(14)	8.799(3)	9.479(2)	424.0(2)
**S-1020**	5.0873(12)	8.795(2)	9.483(2)	424.3(2)
**S-1032**	5.0875(13)	8.794(2)	9.481(2)	424.2(2)
**S-1044**	5.0865(10)	8.796(2)	9.478(1)	424.0(1)
**S-1061**	5.0865(9)	8.791(2)	9.482(1)	424.0(1)
**S-1063**	5.0870(14)	8.791(3)	9.483(2)	424.1(2)
**S-1104**	5.0882(8)	8.792(1)	9.4772(8)	424.0(1)
**S-1142**	5.0927(5)	8.786(1)	9.4706(9)	423.8(1)
**S-1198**	5.0932(6)	8.786(1)	9.4700(9)	423.8(1)
**S-1213**	5.0928(3)	8.786(1)	9.4698(4)	423.7(1)

§ 2. Morphology

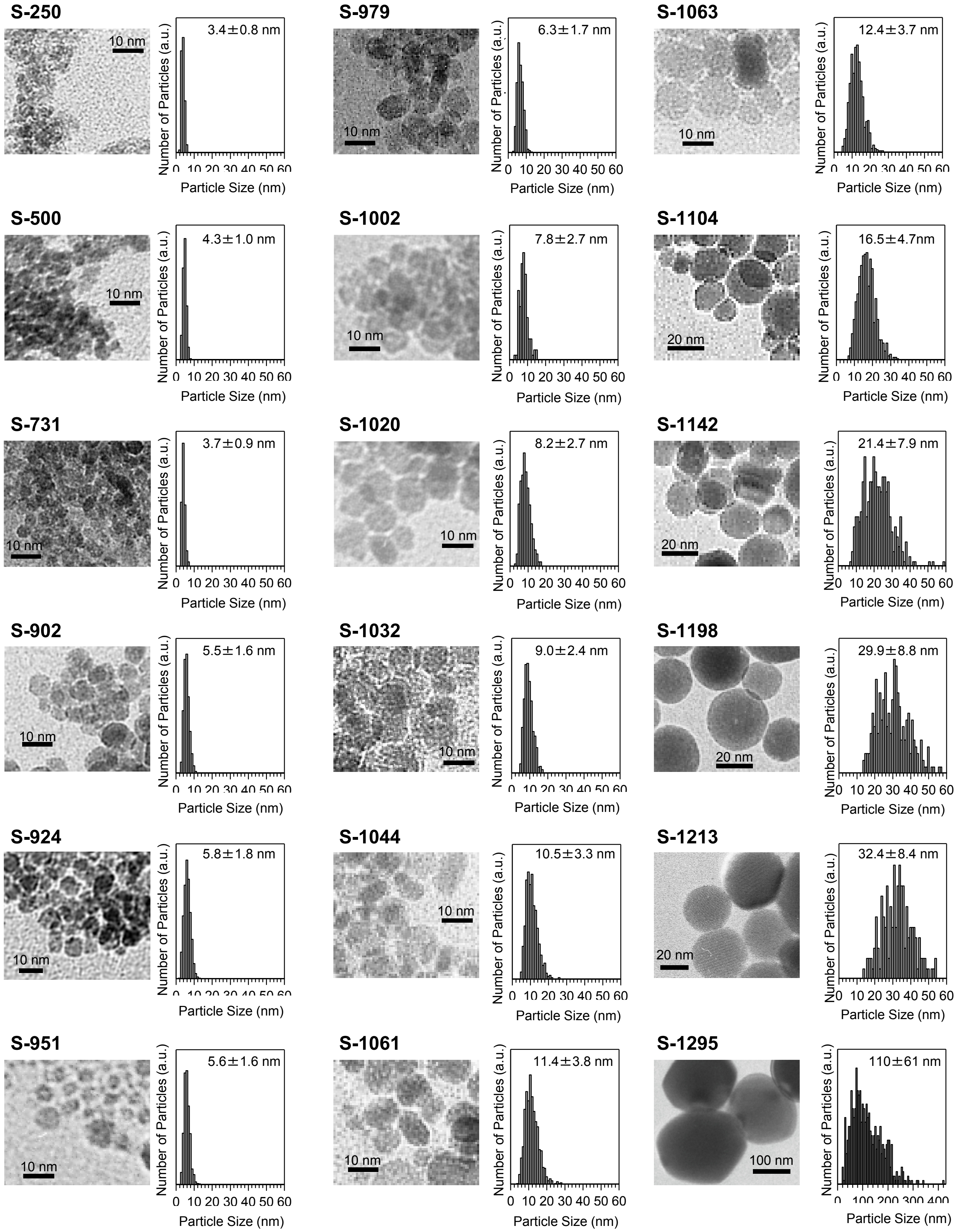

Figure S2. TEM images and particle size distributions for **S-250**, **S-500**, **S-731**, **S-902**, **S-924**, **S-951**, **S-979**, **S-1002**, **S-1020**, **S-1032**, **S-1044**, **S-1061**, **S-1063**, **S-1104**, **S-1142**, **S-1198**, **S-1213**, and **S-1295**.

§ 3. Magnetic hysteresis loops

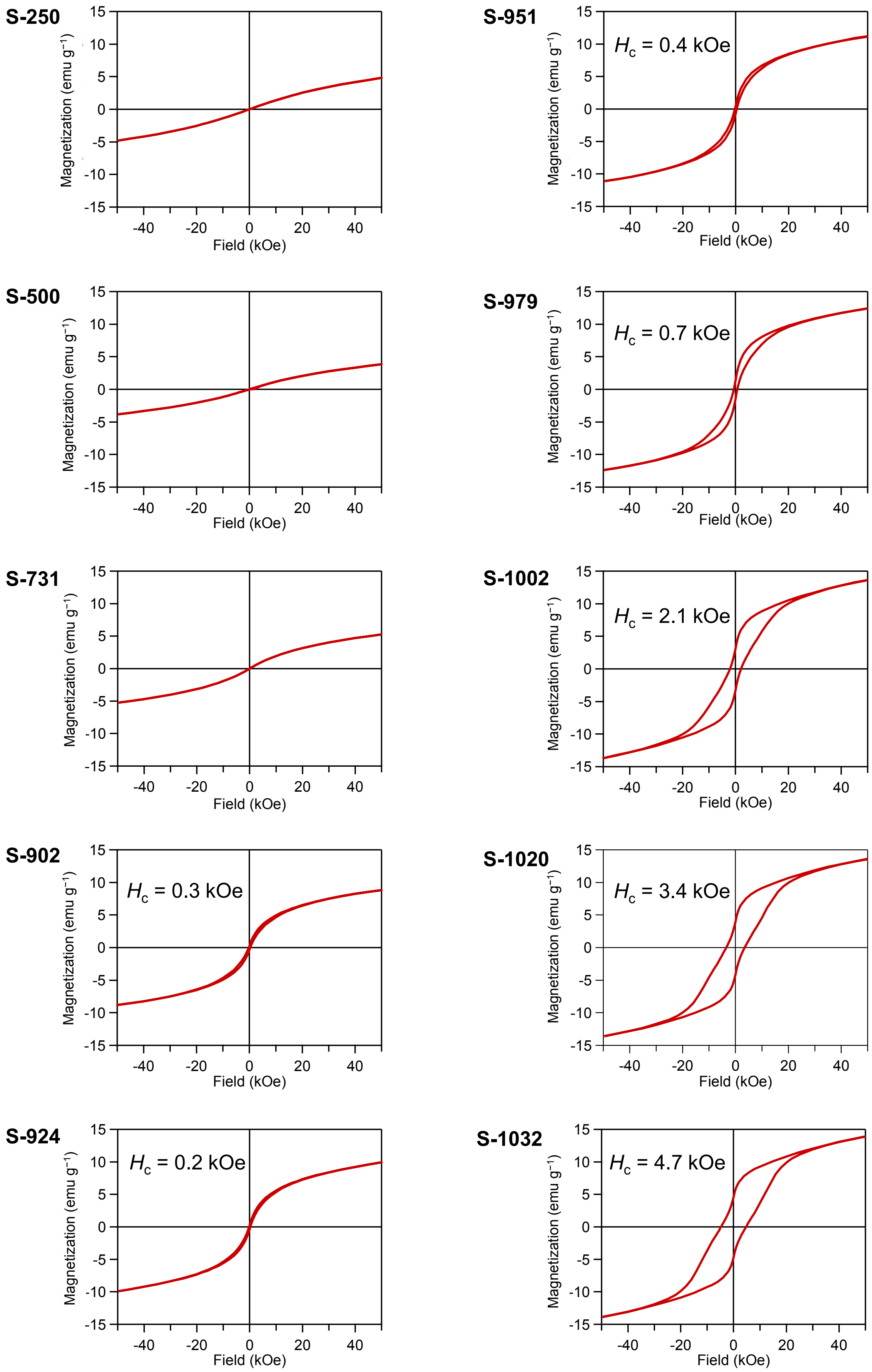

Figure S3-1. Magnetic hysteresis loops measured at 300 K for **S-250**, **S-500**, **S-731**, **S-902**, **S-924**, **S-951**, **S-979**, **S-1002**, **S-1020**, and **S-1032**.

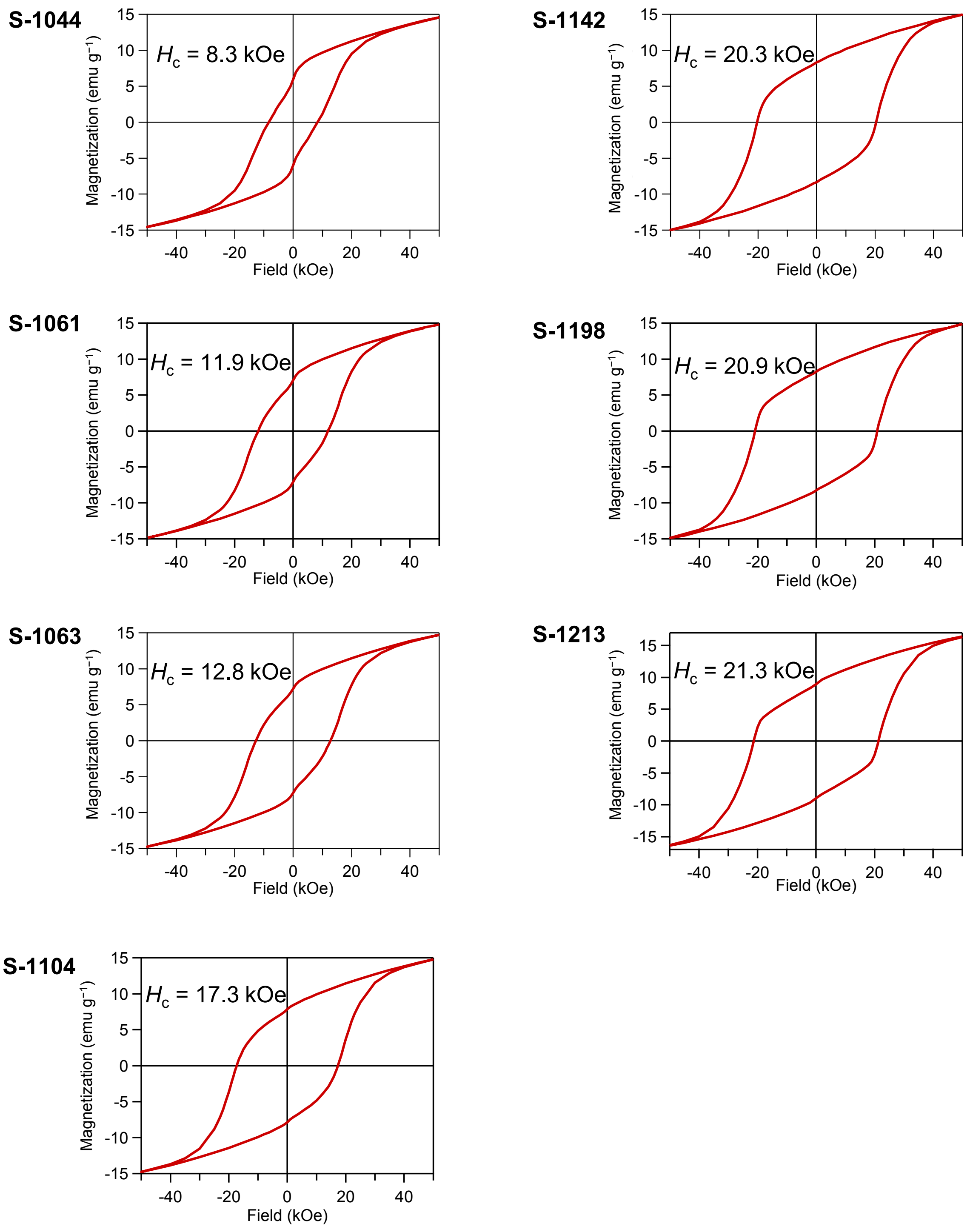

Figure S3-2. Magnetic hysteresis loops measured at 300 K for **S-1044**, **S-1061**, **S-1063**, **S-1104**, **S-1142**, **S-1198**, and **S-1213**.

§ 4. Magnetization versus temperature curves

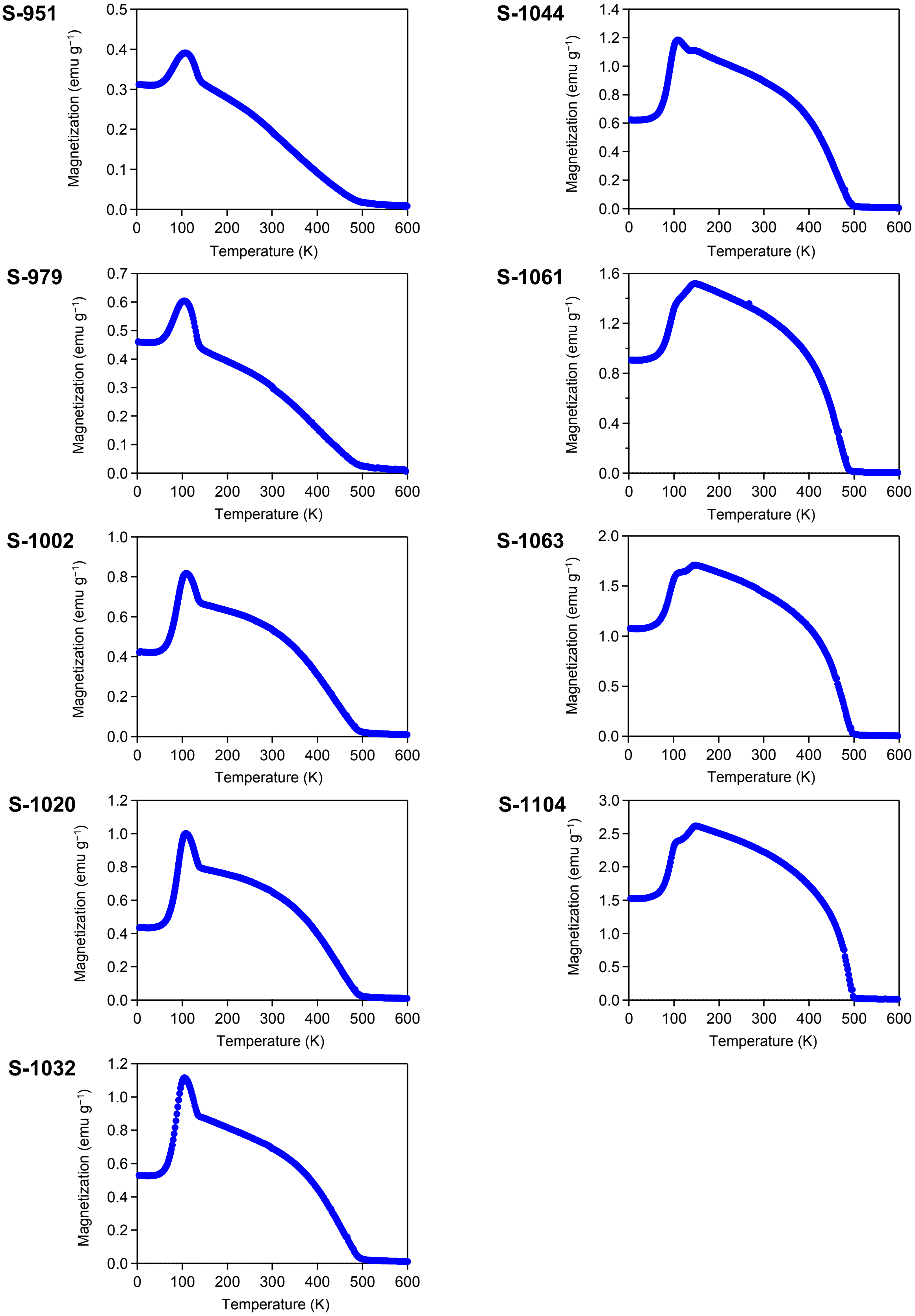

Figure S4. Field-cooled magnetization (FCM) curves of pure ε-Fe_2O_3 samples (**S-951**, **S-979**, **S-1002**, **S-1020**, **S-1032**, **S-1044**, **S-1061**, **S-1063**, and **S-1104**) measured under 100 Oe.

§ 5. Particle size dependences of the H_c values

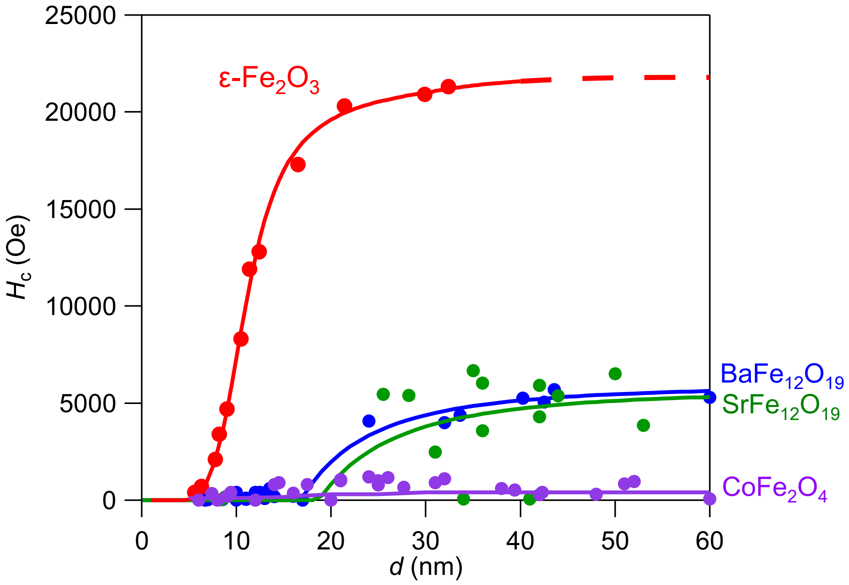

Figure S5. Particle size (*d*) dependences of the H_c values. H_c versus *d* plots for ε-Fe_2O_3 (red), $BaFe_{12}O_{19}$ (blue), $SrFe_{12}O_{19}$ (green), and $CoFe_2O_4$ (purple). Lines are eye guides which are drawn based on the *d* dependence equation of H_c.

References:

$BaFe_{12}O_{19}$

[S1] Kolev, S. & Koutzarova, T. *J. Phys. Conf. Ser.* **514**, 012021 (2014). [S2] Müller, R., Hergt, R., Dutz, S., Zeisberger, M. & Gawalek, W. *J. Phys.: Condens. Mat.* **18**, S2527 (2006). [S3] Sudakar, C., Subbanna, G. N. & Kutty, T. R. N. *J. Electroceram.* **6**, 123 (2001). [S4] Duong, G. V. et al. *J. Non-Cryst. Solids* **353**, 811 (2007). [S5] Santos, J. V. A., Macedo, M. A., Cunha, F., Sasaki, J. M. & Duque, J. G. S. *Microelectr. J.* **34**, 565 (2003). [S6] Gunes, S., Akay, S. K. & Kara, A. *Acta Phys. Pol. A* **125**, 538 (2014). [S7] Shang, H., Wang, J. & Liu, Q. *Mater. Sci. Eng. A* **456**, 130 (2007). [S8] Dhage, V. N., Mane, M. L., Keche, A. P., Birajdar, C. T. & Jadhav, K. M. *Physica B* **406**, 789 (2011). [S9] Fang, Q.-Q., Zhong, W. & Du, Y.-W. *Chin. Phys. Lett.* **16**, 285 (1999). [S10] Krishna murthy, J., Mitra, C., Ram, S. & Venimadhav, A. *J. Alloy. Compd.* **545**, 225 (2012). [S11] Jalli, J. et al. *IEEE Trans. Magn.* **45**, 3590 (2009). [S12] Li, Y., Wang, Q. & Yang, H. *Curr. Appl. Phys.* **9**, 1375 (2009).

$SrFe_{12}O_{19}$

[S13] Shinde, S. R. et al. *J. Appl. Phys.* **88**, 1566 (2000). [S14] Yang, X., Li, Q., Zhao, J., Li, B. & Wang, Y. *J. Alloy. Compd.* **475**, 312 (2009). [S15] Chen, D.-H. & Chen, Y.-Y. *Mater. Res. Bull.* **37**, 801 (2002). [S16] Choy, J.-H., Han, Y.-S. & Song, S.-W. *Mater. Lett.* **19**, 257 (1994). [S17] Hessien, M. M., Rashad, M. M. & El-Barawy, K. *J. Magn. Magn. Mater.* **320**, 336 (2008). [S18] Masoudpanah, S. M. & Seyyed Ebrahimi, S. A. *J. Magn. Magn. Mater.* **323**, 2643 (2011). [S19] Lee, S. W., An, S. Y., Kim, S. J., Shim, I.-B. & Kim, C. S. *IEEE Trans. Magn.* **39**, 2899 (2003). [S20] Li, X., Lu, G. & Li, S. *J. Mater. Sci. Lett.* **15**, 397 (1996).

$CoFe_2O_4$

[S21] Maaz, K., Mumtaz, A., Hasanain, S. K. & Ceylan, A. *J. Magn. Magn. Mater.* **308**, 289 (2007). [S22] Rajendran, M. et al. *J. Magn. Magn. Mater.* **232**, 71 (2001). [S23] Kumar, V., Rana, A., Yadav, M. S. & Pant, R. P. *J. Magn. Magn. Mater.* **320**, 1729 (2008). [S24] El-Okr, M. M. et al. *J. Magn. Magn. Mater.* **323**, 920 (2011). [S25] Panda, R. N., Shih, J. C. & Chin, T. S. *J. Magn. Magn. Mater.* **257**, 79 (2003). [S26] Singhal, S., Barthwal, S. K. & Chandra, K. *J. Magn. Magn. Mater.* **306**, 233 (2006).

§ 6. Crystallographically oriented nanocrystal film

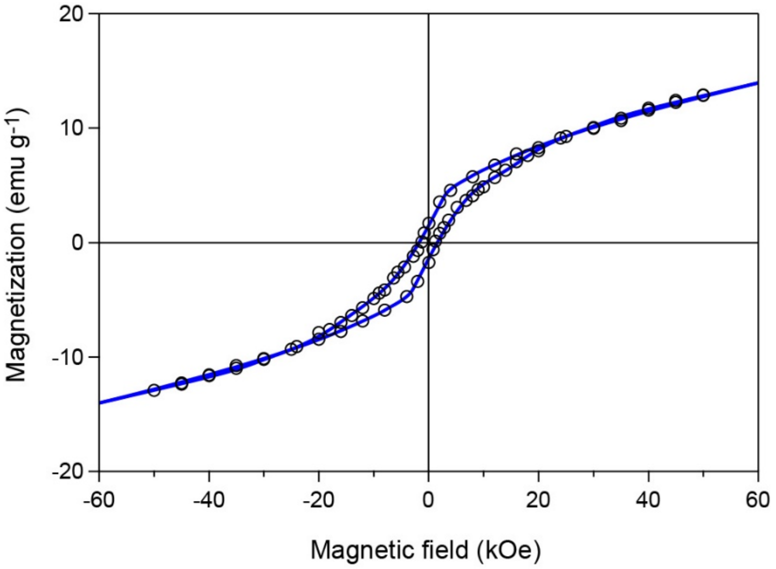

Figure S6. The magnetic hysteresis loop of the crystallographically oriented **S-1020** nanocrystal film measured in the applied magnetic field perpendicular to the easy-axis at 300 K. The blue line is a guide to the eye.

§ 7. SHG measurement

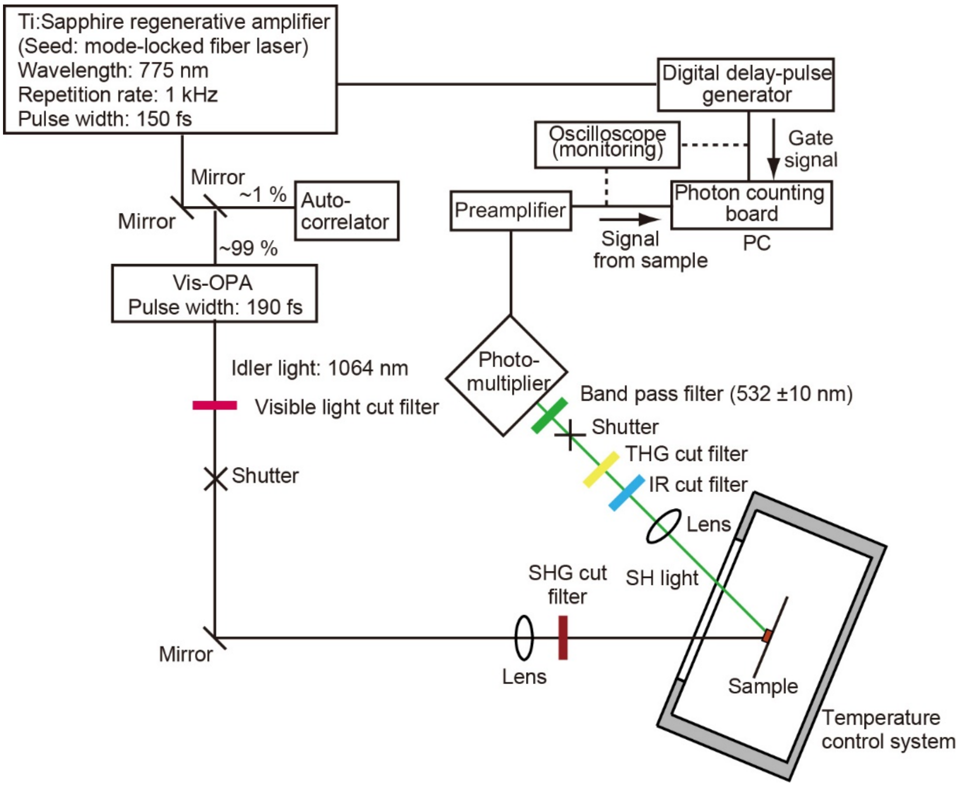

Figure S7. The experimental setup for SHG measurement.
